# Outcome of polymerase chain reaction (PCR) analysis in 100 suspected cases of infectious uveitis

**DOI:** 10.1186/s12348-017-0144-1

**Published:** 2018-01-10

**Authors:** Ranju Kharel (Sitaula), M. K. Janani, H. N. Madhavan, Jyotirmay Biswas

**Affiliations:** 1 Department of Ophthalmology, Maharajgunj Medical Campus, B. P. Koirala Lions Centre for Ophthalmic Studies, Institute of Medicine, Tribhuvan University, Maharajgunj, Kathmandu, Nepal; 2 Sankara Nethralaya Referral Laboratory, Chennai, India; 3 Vidyasagar Institute of Medical Biotechnology and Science, Sankara Nethralaya Referral Clinical Laboratory, Medical Research Foundation, L & T Microbiology Research Centre, 18 College Road, Chennai, 600006 India; 4 Uveitis and Ocular Pathology Department, Medical and Vision Research Foundations, Sankara Nethralaya, College road, Chennai, Tamil Nadu India

**Keywords:** Infection, Ocular fluid, Polymerase chain reaction, Tuberculosis, Uveitis

## Abstract

**Background:**

Polymerase chain reaction (PCR) analysis is an important tool in the diagnosis of infectious uveitis. A retrospective, interventional study of PCR analysis of ocular fluid in suspected infectious uveitis cases between January 2014 to July 2016 was done. Nested, real-time and broad range PCR was performed for detection of the genome of *Mycobacterium tuberculosis*, herpes virus family, Chikungunya virus, *Toxoplasma gondii*, fungus, eubacterium and *propionibacterium acne*.

**Results:**

Total of 100 cases included, mean age was 39.2 ± 15.4 years. Uveitis was unilateral in 82% and granulomatous in 40%. Mean visual acuity at the initial visit and final visit was 0.73 logMar and 0.63 logMar respectively. PCR analysis confirmed the clinical diagnosis in 70.1% patients. The sensitivity, specificity, positive predictive value and negative predictive value of PCR analysis was 90.2%, 93.9%, 93.9% and 90.2% respectively. The quantitative value of real-time *M. tb*. Positive PCR ranged from 32c/ml to 2722 c/ml.

**Conclusions:**

PCR assay is an accurate technique with high sensitivity and specificity to diagnose the DNA genome in infectious uveitis.

## Background

The prevalence of infectious uveitis in India is reported to be as high as 31% [1]. Visual impairment and blindness due to infectious uveitis can be prevented by early identification of the responsible pathogen and the subsequent prompt administration of appropriate antimicrobial therapy. The polymerase chain reaction (PCR) analysis of ocular fluid samples allows early, accurate, and rapid detection of small quantities of DNA or RNA from potential pathogens infecting the uveal tract and is a sensitive and specific method to detect microbial DNA in ocular samples from immunocompetent and immunocompromised patients with uveitis [2–7].

Patients with uveitis of infectious and non-infectious etiologies often share similar clinical signs and symptoms at presentation, representing diagnostic challenges, especially when serology is unable to establish the diagnosis [8]. Having an early definitive laboratory-proven diagnosis is very advantageous in starting timely appropriate effective treatment. So, we investigated the diagnostic utility of real-time and nested PCR samples obtained from the ocular fluid in clinically suspected infectious uveitis. We analyzed the sensitivity, specificity and, predictive values of PCR to detect the etiological agent from aqueous and vitreous humors and compared with the diagnostic hypothesis.

## Methods

A retrospective, interventional study of PCR analysis of ocular fluid in suspected infectious uveitis cases was conducted in January 2014 to July 2016 at Sankara Nethralaya, a tertiary level referral eye center of South India. A total of 122 uveitis cases evaluated during the study period were identified, but 22 cases had incomplete documentation and so were excluded.

Detailed ocular and systemic history and complete eye examination was performed. Snellen VA was converted to logarithm of the minimum angle of resolution (logMar) units for analysis. The tailored laboratory investigations were performed as needed to support the diagnosis. Demographic profile presenting visual acuity and final visual acuity after treatment was noted.

The uveitis cases suspected of infectious origin were placed for aqueous or vitreous tap for PCR analysis. Nested PCR for detection of human herpes simplex virus (HSV1 and 2), varicella zoster virus (VZV), cytomegalovirus (CMV), *Propionibacterium acnes*, *Toxoplasma gondii*, *Mycobacterium tuberculosis*, eubacterial genome, and panfungal genome was performed. And real-time PCR for detection of *Mycobacterium tuberculosis* was performed where tuberculosis DNA detection was done using MPB64 and IS6110 genome. Based on the phonotypic appearance, the appropriate treatment was commended pending the results of PCR testing. When indicated by the subsequent PCR results, the treatment was changed.

All works were performed in an ISO-15189-accredited molecular laboratory. PCR results were reported as detected or not detected within 48–72 h. The diagnosis was called “confirmed” if the PCR analysis detected the same microbes suspected before the PCR analysis, “altered” if the genome other than suspected was identified, and “negative” if any microbial genome not identified.

### Processing of clinical samples

Aqueous humor (AH) samples (150–200 μl) were collected aseptically in a tuberculin syringe with a 30-gauge needle, under aseptic precautions by a single ophthalmologist as an outpatient department (OPD) procedure, and undiluted vitreous humor (VH) samples were obtained by a 23-gauge needle pars plana vitrectomy in an operation theater. The samples were transferred onto pre-sterilized microfuge tubes and stored at − 20 °C for DNA extraction.

### DNA extraction

Leukocytes of the buffy coat suspended in 100 μl of aqueous were subjected for DNA extraction following the manufacturer’s instructions of QIAGEN DNA extraction kit, Hilden, Germany.

### Polymerase chain reaction for detection of infectious agents

PCR testing was performed for the commonest causative organisms, namely, CMV, HSV type1 and type2, VZV, *T*. *gondii*, *M*. *tuberculosis*, *P*. *acnes*, Eubacterium, and Panfungus genome using previously published primer sequences.

To detect the DNA for bacterial species and fungus, broad-range PCRs were performed targeting16S ribosomal DNA and 18S ribosomal DNA, respectively, in accordance with our previously reported methodology.

The PCR mixture (50 μl) contained 100 mM of dNTP mixture, 10× PCR buffer with 15 mM MgCl2, 1 μM of each forward and reverse primer, and 3 U/μl Taq DNA polymerase. Ten microliters of extracted positive control or test sample DNA was added to the first round PCR reaction mixture. For the second round amplification, 5 μl of the first round product was added to the 50 μl of the PCR mix containing 10 mM of each dNTP, 10× buffer, 1 μM of each forward and reverse primer, and Tag DNA polymerase. Two controls (one reagent control and another one serves as reaction control) were included in each PCR run. The PCR results were considered valid only when the reagent controls were negative and the specific amplified product was obtained with the positive controls. To prevent contamination DNA extraction, PCR cocktail preparation, amplification, and analysis of results were carried out in physically separated rooms. The *M*. *tuberculosis* load was estimated in the DNA extracts of test samples using a commercial kit—Geno-sen’s *M*. *tuberculosis* Real Time PCR kit (Genome Diagnostics Pvt. Ltd)—and the assay was performed on Rotor Gene (Hilden, Germany) real-time PCR equipment based on Taqman principle.

### Positive controls

DNA was extracted from *Mycobacterium tuberculosis* strain H37Rv, herpes simplex virus (HSV) 1—ATCC VR 733, herpes simplex virus (HSV) 2 ATCC 753167, cytomegalovirus (CMV)—ATCC 169, varicella zoster virus (VZV)—ATCC Oca strain, *Toxoplasma gondii*— ATCC 50869, Eubacteria (*Propionibacterium acnes* lab isolate), and fungus (*Candida albicans*) ATCC 90028. All the standard strains are maintained in the laboratory.

### Detection of amplified products

Visualization of PCR product was done by subjecting 10 μl of amplified reaction mixture to electrophoresis on a 2% agarose gel incorporating 5 μg ml of ethidium bromide in 1× Tris-Borate buffer (pH 8.2–8.6) and documented on gel documentation system (Vilber Lourmat, France).

Initial pre-PCR diagnoses were established on the basis of history, clinical findings, and investigation results. The major outcomes considered were the correlation of the pre-PCR diagnosis with the PCR results and change in treatment modality. The sensitivity and specificity of PCR was calculated based on the final diagnoses derived from the clinical course response to treatment and results of ancillary investigations. Positive predictive value (PPV) and negative predictive value (NPV) for PCR testing were also calculated and derived using Bayes’ theorem.

The treatment consisted of anti-tubercular therapy (ATT) for 9 months for ocular tuberculosis, intravenous acyclovir, or oral valacyclovir for 90 days in HSV or VZV retinitis. Oral valgancyclovir was considered for CMV retinitis and oral clindamycin 6 weeks in ocular toxoplasmosis and broad-range antimicrobials in endophthalmitis. Oral corticosteroid was considered whenever required.

Approval from institution ethics committee was taken and adherence to the tenets of Declaration of Helsinki maintained. Descriptive statistics were computed and statistical software (SPSS 14) was used for univariate and multivariate analysis and for the logistic regression analysis; *p* value < 0.05 was considered to be significant. The 95% bootstrapped confidence intervals were computed for all summary statistics. A chi-square test and paired *t* test was used to compare sensitivity values. Stepwise regression method was used to determine the significant predictors for final visual acuity. Then, multiple regression models were used to predict final visual acuity with different types of uveitis.

## Results

A total of 100 uveitis patients who underwent ocular fluid PCR analysis were included in the study.

### Demographic profile

The mean age was 39.2 ± 15.4 years, the range being 9–72 years. The male were 52% and female were 48%. There was unilateral involvement in 82% and bilateral in 18% of the cases. The demographics and the clinical features of the patients are shown in Table 1.

**Table 1:** Demographics profile and clinical features

Age	Mean	39.2 ± 15.4 years
	Range	9–72 years
Gender	Male	52%
	Female	48%
	Ratio	1.08
Affected eye	Unilateral	82%
	Bilateral	18%
Site of PCR	Aqueous tap	98%
	Vitreous tap	2%
Mean presenting visual acuity	0.73 logMar unit	
Keratic precipitates	Fine KPs	36%
	Mutton fat KPs	18%
	No KPs	50%
Anterior chamber cells	Present	56%
	Absent	44%
Vitreous cells	Present	46%
	Absent	54%
According to anatomical site	Anterior uveitis	34%
	Intermediate uveitis	17%
	Posterior uveitis	38%
	Panuveitis	4%
	Anterior + intermediate uveitis	7%
According to duration	Acute uveitis	37%
	Chronic uveitis	30%
	Recurrent uveitis	33%
According to pathology	Granulomatous uveitis	40%
	Non-granulomatous uveitis	60%
According to anatomical site	Anterior uveitis	34%
	Intermediate uveitis	17%
	Posterior uveitis	38%
	Panuveitis	4%
	Anterior + intermediate uveitis	7%

Mean visual acuity at presentation was 0.73 logMar unit and the mean intraocular pressure was 15.56 ± 3.76 mmHg. Among these 100 cases subjected for ocular fluid PCR analysis, posterior uveitis was the most prevalent type (38%) followed by anterior uveitis (34%).The classification of various types of uveitis are given in Table 1. There were 37% patients who had an acute episode of uveitis, 30% with chronic uveitis, and recurrent uveitis in 33%.

### Initial clinical diagnoses

Initial pre-PCR diagnoses were established based on the history, clinical findings, and investigation results. The commonest type of uveitis subjected for PCR was posterior uveitis (38%). The commonest suspected infectious etiology was tuberculosis (37.1%) followed by viral uveitis (23.8%) which is shown in Table 2.

**Table 2:** Correlation of pre-PCR diagnosis with post-PCR results

Pre-PCR diagnoses	No.	MPB64/IS6110	HSV 1	HSV 2	VZV	CMV	*T*. *gondii*	*P*. *acnes*	Eubacterium	Panfungus	Chikunguniya	Positive PCR	
												No.	Percentage
Tubercular uveitis	39	28										28	71.8
Acute retinal necrosis	23		3	2	7							14	60.8
Chronic post op endophthalmitis	9							2	4	3		9	100
Toxoplasmosis	5						0					0	0
CMV retinitis	2					2						2	100
Chikunguniya uveitis	1										1	1	100
Non-specific etiology	21	1	2		1							5	23.8
TOTAL	100	29	5	2	8	2	0	2	4	3	1	70	100

### PCR results

**Fig. 1:**
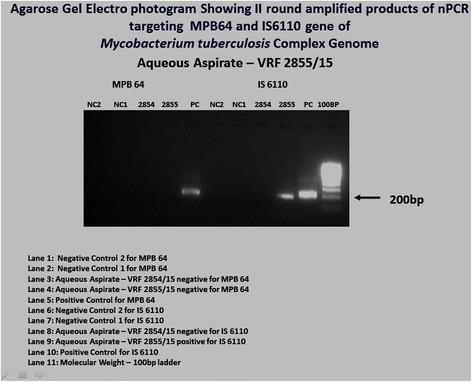
Agarose gel electophotogram showing the detection of *Mycobacterium tuberculosis* complex genome

**Fig. 2:**
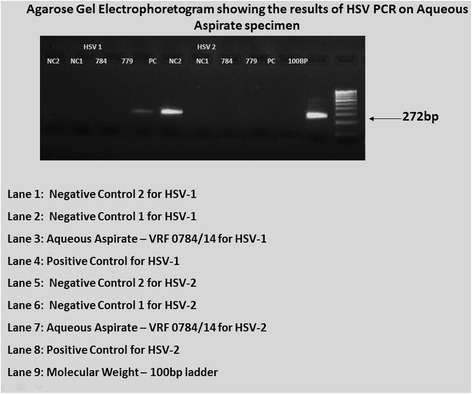
Agarose gel electophotogram showing the detection of herpes simplex virus (HSV) genome

**Fig. 3:**
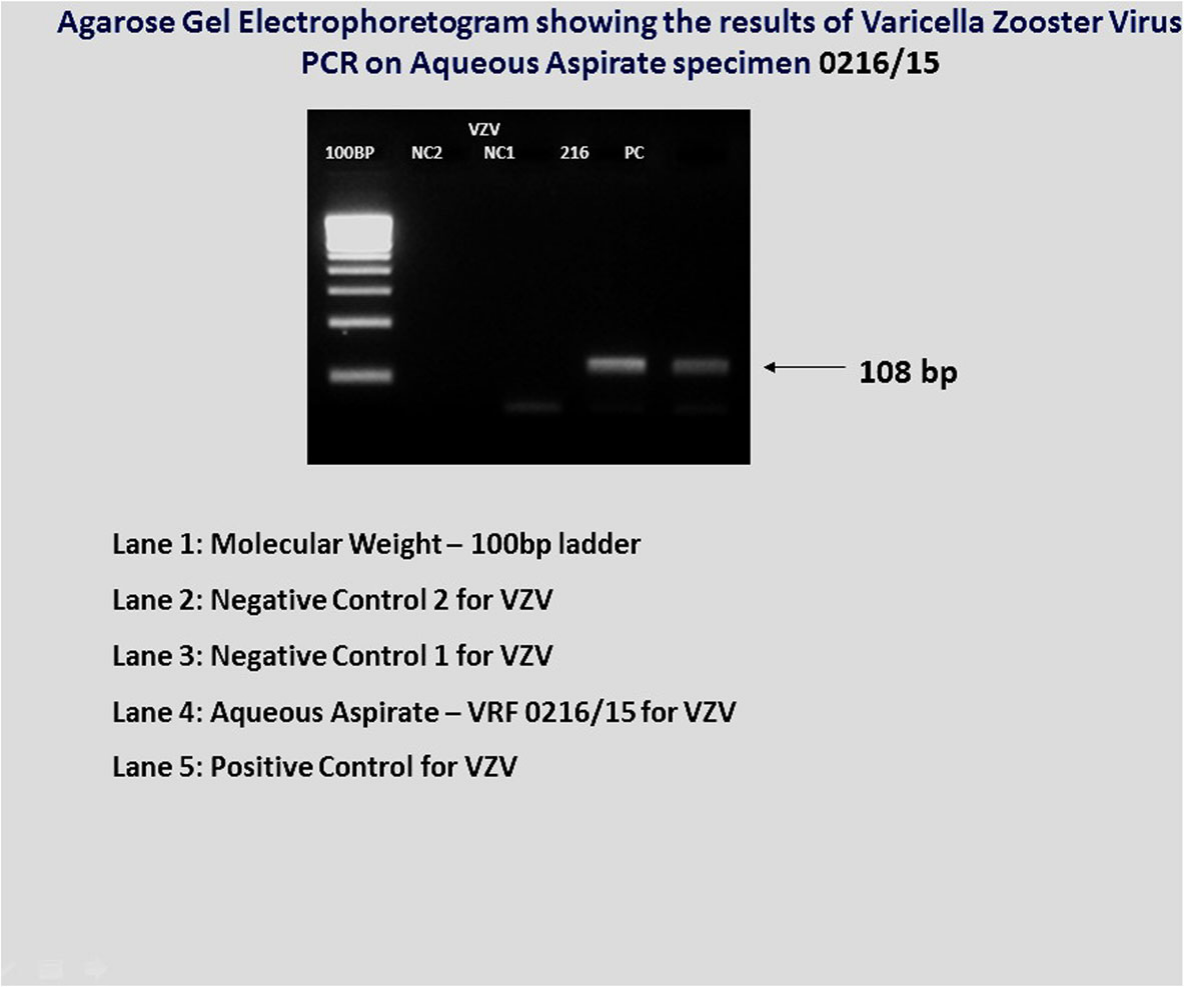
Agarose gel electophotogram showing the detection of varicella zoster virus (VZV) genome

**Fig. 4:**
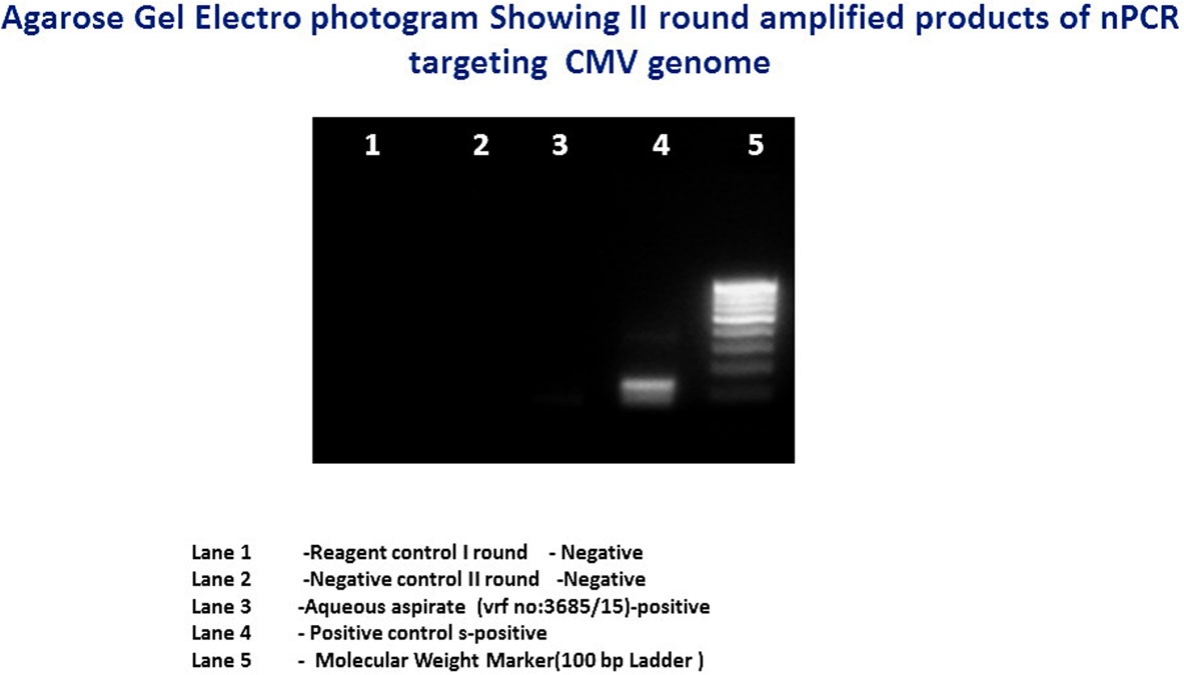
Agarose gel electophotogram showing the detection of cytomegalovirus (CMV) genome

Among 100 eyes of 100 patients, PCR analysis confirmed the initial clinical diagnosis in 70.1% patients. The correlation of pre- and post-PCR diagnoses is depicted in Table 2. But PCR altered our treatment in 17.7%. The overall sensitivity and specificity of PCR analysis of ocular fluid was found to be 90.2 and 93.9%, respectively. The PPV, defined as the likelihood of having disease related to the tested infectious agent given positive PCR results, was 93.9% and NPV, defined as the likelihood of not having the specified disease given negative PCR results, was 90.2% in the overall analysis (Table 3). The sensitivity, specificity, and predictive values of the individual organism were also analyzed (Table 4) and the agarose gel electrophotogram of detection of various DNA genomes shown in Figs. 1, 2, 3, and 4. Real-time PCR was done for *M*. *tuberculosis* and this showed the quantitative value ranging from 32 to 2722 c/ml.

**Table 3:** The sensitivity, specificity, and predictive values of PCR test

Parameters	Value (%)	95% confidence interval
Overall sensitivity	90.2	78.59 to 96.74%
Overall specificity	93.9	83.13 to 98.72%
Positive predictive value	93.9	83.13 to 98.72%
Negative predictive value	90.2	78.59 to 96.74%

**Table 4:** Individual organism sensitivity, specificity, and predictive value

Organisms	Sensitivity (%)	Specificity (%)	Positive predictive value (%)	Negative predictive value (%)
Mycobacterium	71.4	76.77	54	98.7
CMV	100	71	15.4	100
HSV 1	100	74.4	23	100
HSV 2	100	71.8	15.4	100
VZV	71.43	78.1	41.7	92.6
*Toxoplasma gondii*	0	66.7	0	100
Fungus	66.67	50	66.7	50
*Propionibacterium acnes*	100	50	50	100
Eubacterial	75	0	75	0

The visual acuity at presentation was correlated with the visual acuity at the final follow-up. Mean visual acuity at initial visit and final visit was 0.73 and 0.63 logMar unit, respectively, using *t* test. Though the vision improved, no significant *p* value change was noted.

The stepwise regression method was used to determine the significant predictors for final visual acuity. Then, multiple regression model was used which revealed intermediate uveitis and panuveitis as important predictors for final visual acuity. The ANOVA test of regression model showed that presence of intermediate uveitis and panuveitis was directly related with decline in final visual acuity by 0.491 + 0.646 logMar unit and 0.491 + 0.983 logMar unit. The coefficient of determination showed R2 = 0.06.9% which meant that only 6.9% of the variation in the dependent variable is explained by the model, while the remaining 93.1% is left unexplained.

The collection of the aqueous fluid was done safely in the outpatient department (OPD) and only one eye had complication in form of hyphema.

## Discussion

This study was conducted to ascribe the pathogen distribution based on ocular fluid PCR testing of patients with suspected infectious uveitis because incorrect diagnosis can lead to potential ocular morbidity and drug-related health hazard [5].

In Japan [9], 66.5% were identified with specific uveitic etiologies consisting of non-infectious diseases (50.1%) and infectious diseases (16.4%). Latest studies from India have shown the prevalence of infectious uveitis to be as high as 31% [1].

As the posterior uvea is the most common location of infection, posterior uveitis is commonly associated with infectious origin, but the anterior uveitis often has idiopathic origin so commonest indication for ocular fluid tap for PCR analysis in our series too was posterior uveitis (38%). However, analysis of aqueous was more commonly performed because anterior chamber tap is a less invasive and hazardous procedure than the vitreous aspiration and can also be performed in the OPD.

The gold standard used in this study was the final clinical diagnosis after consideration of all data, including the PCR results and response to treatment. With the help of PCR, initial clinical diagnosis was confirmed in 70.1% but was altered in 17.7% after PCR testing. The initial pre-PCR clinical diagnosis was uncertain in 21 cases. This was due to difficulty in diagnosis in presence of posterior synechiae and significant vitritis. Among these, the PCR confirmed the diagnosis in five cases (23.8%).

Using comprehensive PCR system, Sugita et al. reported the sensitivity, specificity, PPV, and NPV of PCR for the diagnosis of infectious ocular diseases as 91.3, 98.8, 98.5, and 92.4%, respectively [10]. Though we had used the uniplex PCR system, the sensitivity was 90.2%, specificity was 93.9%, PPV was 93.9%, and NPV was 90.2%. These results are very comparable and shows that the uniplex PCR performed in a standardized manner can yield as reliable results as comprehensive or multiplex PCR.

Our sensitivity and specificity results were comparable to Harper et al.’s study where the PCR analysis for posterior segment infectious uveitis had sensitivity of 81% and specificity of 97% [11]. But NPV in their study [12] was low (68%) compared to ours. PPV and NPV are primarily dependent on the prevalence of disease; therefore, these numbers can vary with the clinical setting.

In some cases, diagnostic vitrectomy will remain the most appropriate diagnostic strategy because it allows access to greater quantities of intraocular fluid and to tissue specimens for cytological analysis, flow cytometry, and retinochoroidal biopsy [12]. However, we could not perform the comparison between the utility of aqueous PCR versus vitreous PCR analysis in our study due to limited number of cases of vitreous biopsy.

PCR has a very low false-positive rate when used on ocular fluids. In fact, false-positive results are possible from contamination and false-negative results are possible from polymorphism, specimen degradation, or failure to sample in the acute stages of disease [13]. But the use of positive and negative controls in our PCR laboratory had reduced the burden of false-positive and false-negative results.

Early in 1999, Arora et al. evaluated the role of PCR for detection of *Mycobacterium tuberculosis* in the aqueous humor samples obtained from eyes with active uveitis and showed that it can be effectively used for the diagnosis of intraocular tuberculosis [14] which was supported by the review report by Gupta et al. [15] As culture of mycobacterium is difficult and time consuming, presently, the use of real-time PCR has helped a lot to establish ocular TB, and our study too proved PCR to be an important tool for rapid detection of the mycobacterial genome in suspected tubercular uveitic cases with a sensitivity of 71.4% and specificity of 76.8% and the quantitative value of real-time *M*. *tuberculosis* positive PCR ranging from 32 to 2722 c/ml.

Necrotizing herpetic retinitis is a sight-threatening ocular emergency where rapid diagnosis and appropriate treatment with sensitive antiviral agents in the early stage of the disease is a must. PCR on aqueous humor sample allowed identification of the causative agent in ARN from 40% [16] to 86.4% [5] and these results were similar to our findings.

The first report of use of PCR in ophthalmology in India was by one of our authors [17] in 1993 for detection of CMV retinitis then after various literature have shown the PCR sensitivity for CMV retinitis to range from 91 to 95% [2, 5, 7, 18], and our present experience showed a sensitivity of 100% and specificity of 71% in CMV retinitis case.

Diagnosis of ocular toxoplasmosis is mainly clinical, and PCR analysis in patients with ocular toxoplasmosis is generally less sensitive than viral retinitis. Studies have shown variable sensitivity ranging from 27 to 85% [2, 19–21]. Reports from Brazil [22] have shown detection of *Toxoplasma gondii* DNA in an aqueous sample with qPCR in 37.2%, but in our scenario, out of five cases placed for PCR for identification of *Toxoplasma gondii*, all of them were negative. This poor sensitivity in detection of *Toxoplasma gondii* in our experience may be due to the inappropriate timing of test as chances of PCR positivity may be higher in early 2 weeks but we at tertiary level are dealing mainly with referred cases with late presentation. Secondly, this could be due to the difference in the strains of the toxoplasma and the use of in-house PCR primer. But we still believe that ocular toxoplasmosis is a clinical diagnosis and needs no laboratory investigations unless it presents in atypical pattern.

Endophthalmitis is caused by either endogenous or exogenous infection of various pathogens, mostly bacteria or fungi. We had used broad-range PCR test (16S and 18S RNA) and out of nine suspected cases of endophthalmitis, the pathogen were detected in all nine cases (Eubacterium in four, Panfungus in three, and *P*. *acnes* in two cases).

The stepwise regression pointed that decline in visual acuity was related in case of intermediate uveitis and panuveitis as compared to anterior and posterior uveitis. The *p* value of *f* test statistic was < 0.05, indicating that the model is a good fit in predicting final visual acuity.

Overall, PCR analysis in our study proved as an important diagnostic tool to establish, alter, or exclude any infectious etiology of uveitis. But correlation between the DNA load and prognosis still needs to be explained with further studies in future.

The major limitations of our study were retrospective, no controls for comparison, and criteria for performing PCR were not well specified. Moreover, our study was not a population-based study but a tertiary level referral center study which could have resulted in possible selection bias. Also, few patients had short-term follow-up and few had drop out to follow-up.

Another limitation was we did not use multiplex PCR so specific primers were used for individual organism. Hence, the results did not make it possible to include or exclude infections as the potential cause of an ocular disorder in a single attempt. However, multiplex PCR cannot quantitatively measure copy number of genomic DNA. So, comprehensive PCR system consisting of a combination of multiplex PCR and real-time PCR has recently been developed for intraocular fluids with the aim to diagnose infectious uveitis.

## Conclusions

Accurate and timely etiological diagnosis is of utmost importance for the management of patients with uveitis. Though PCR testing of ocular fluid is an invasive procedure, its role in identifying the infectious etiology in uveitis disorders is remarkable, hence should be considered as an adjunct test in any suspected infectious uveitis.
